# Circulating Betatrophin Levels and Gestational Diabetes Mellitus: A Systematic Review and Meta-Analysis

**DOI:** 10.1371/journal.pone.0169941

**Published:** 2017-01-12

**Authors:** Fei-Juan Kong, Lei-Lei Ma, Ge Li, Yi-Xin Chen, Jia-Qiang Zhou

**Affiliations:** 1 Department of Endocrinology, Sir Run Run Shaw Hospital, School of Medicine, Zhejiang University, Hangzhou, China; 2 Department of Critical Care Medicine, Zhejiang Provincial People's Hospital, Hangzhou, China; 3 Department of Cardiology, Shanghai Institute of Cardiovascular Diseases, Zhongshan Hospital, Fudan University, Shanghai, China; Shanghai Diabetes Institute, CHINA

## Abstract

**Objective:**

The association between circulating betatrophin levels and gestational diabetes mellitus (GDM) is controversial. The aim of our study was to systematically review available literature linking betatrophin to GDM for a comprehensive understanding of the relationship between circulating betatrophin levels and GDM in human.

**Methods:**

PubMed, The Cochrane Library, Medline and CNKI were searched for studies published up to August 2016. Manual searches of references of the relevant original studies were conducted. Pooled estimates were measured using the fixed or random effect model. Overall effect was reported in a standard mean difference (SMD). All data were analyzed with Review Manager 5.3 and Stata 12.0.

**Results:**

Of 25 references reviewed, 8 studies met our inclusion criteria and contributed to meta-analysis. All the studies were used to evaluate the relationship between betatrophin levels in blood and GDM. Betatrophin levels were significantly elevated in women with GDM compared with those without GDM (SMD = 1.05; 95% CI: 0.41–1.68, P = 0.001). This evidence was more consistent among women with betatrophin blood draw during the third trimester (SMD = 1.3, 95% CI: 1–1.61, P < 0.001) and for women BMI ≥ 28 kg/m^2^ (SMD = 1.53, 95% CI: 1.30–1.75, P < 0.001).

**Conclusions:**

The evidences from this meta-analysis indicated that the levels of circulating betatrophin were significantly elevated among women with GDM compared with women with normal glucose tolerance, especially with BMI ≥ 28 kg/m^2^ and in the third trimester.

## Introduction

Gestational diabetes mellitus (GDM), one of the most common pregnancy complications, is described as impaired glucose tolerance that begins or is initially recognized during pregnancy. As patients suffering from GDM usually do not present any clinical symptom, GDM screening has become a routine prenatal project during the second trimester of pregnancy nowadays. During the last few decades, GDM affects up to 14% of all pregnancies depending on different diagnostic criteria and ethnic origin [1]. GDM not only increases maternal incidence of type 2 diabetes mellitus (T2DM) and metabolic syndrome at follow-up, but also will be associated with various adverse acute outcomes and long-term metabolic derangements in offspring [2–5]. However, the underline mechanisms of GDM remain unclear.

Betatrophin, a newly identified circulatory hormone, which is predominantly secreted by liver and adipose tissue in mice, but primarily expressed in the liver in humans, has been reported to be involved in glucose and lipid metabolism [6–8]. Betatrophin is attracting increasing attention due to its role in promoting pancreatic β-cell proliferation and improving glucose tolerance. Recent study has suggested that overexpression of betatrophin in mouse liver led to an elevated proliferation of pancreatic β-cell and a compensatory expansion of β-cell mass in an insulin resistance mouse model induced by insulin receptor antagonist S961 [9, 10]. Moreover, observational studies in human beings also demonstrated that the alteration of betatrophin was linked to several health conditions, including obesity, T2DM [11, 12] and GDM [13–20]. The scientific evidence linking betatrophin with T2DM or obesity is growing large, but data investigating the correlation between betatrophin status and GDM are controversial. In this regard, some studies reported that women with GDM showed higher concentrations of betatrophin compared to healthy pregnant women [13–18, 20], while Huang *et al* showed the contrary [19]. Whereas the potential causes of these conflicting results were poorly described.

In order to provide a more comprehensive estimation of the association between betatrophin levels in blood and GDM, we performed a systematic review and meta-analysis on related studies aiming for getting a more persuasive conclusion.

## Methods

Our review followed the Meta-Analyses and Systematic Reviews of Observational Studies (MOOSE) guidelines [21]. The data were presented according to the recommendations of the PRISMA statement [22].

### Search strategy

A systematic search of studies was performed on the association of betatrophin levels and GDM in the published databases in English of PubMed, The Cochrane Library, Medline and in Chinese of CNKI (Chinese National Knowledge Infrastructure) up to August 2016. The search strategy included key terms that were summarized as follows: “betatrophin”, “ANGPTL8”, “lipasin”, “C19ORF80”, “TD26”, “RIFL”, “gestational diabetes mellitus”, “GDM”, “diabetes pregnancy”, “insulin gestation”. References from these relevant studies were manually searched.

### Inclusion and exclusion criteria

Studies were considered eligible if they met the following criteria: (1) case-control studies comparing circulating betatrophin levels in GDM women and healthy pregnant controls; (2) all participants did not have a previous history of diabetes or present pregnant complications; (3) full-text articles were published in English or Chinese. Studies were excluded if they were (1) available only as abstracts, review studies, case reports, expert comment, or editor opinion, (2) experimentation on animals or in vitro; (3) predefined outcome data required for analyses were lacking.

### Data extraction and quality evaluation

Two reviewers (FJ Kong and LL Ma) independently reviewed all searched studies and extracted data using a predefined form. If there was a discrepancy, a discussion was carried out to reach an agreement. If a consensus could not be reached, a third experienced investigator (JQ Zhou) was consulted. The following information of each study was recorded: first author, year of publication, country of the study, sample source, assay method of betatrophin, sample size of the case and control group, mean and standard deviation (SD) (part of the data were converted) of betatrophin levels, trimester of betatrophin level measurement, and mean and SD of age and body mass index (BMI) of GDM women.

The individual study quality was assessed according to the Cochrane collaboration’s tool for risk of bias, which contains random sequence generation, allocation concealment, blindness, incomplete outcome data, selective outcome reporting, and other biases.

### Statistical analysis

Standard mean difference (SMD) and 95% confidence interval (95% CI) were calculated to assess the differences in betatrophin levels between groups. Significance levels were determined by Z test. Forest plots were used to demonstrate effect sizes and their CI. Heterogeneity amongst the included studies was assessed by Cochran’s Q statistics and I^2^ statistics. According to heterogeneity inspection results, corresponding pooled method was chosen: if I^2^ > 50%, random effect model was used; while I^2^ ≤ 50%, fixed effect model was adapted. We also did subgroup analyses to explore the potential source of heterogeneity if heterogeneity across studies was statistically significant. Potential publication bias was evaluated using Begg’s test and Egger’s test. Sensitivity analysis was carried out by sequentially omitting one single study each time to test the robustness of uncertainty in the meta-analysis. All data were analyzed with Review Manager (RevMan 5.3) statistical software provided by The Cochrane Collaboration and Stata 12.0 (Stata Corp, College Station, TX, USA). The significance level was set as 0.05, except Cochran’s Q test for heterogeneity as 0.1.

## Results

### Literature search

**Fig 1:**
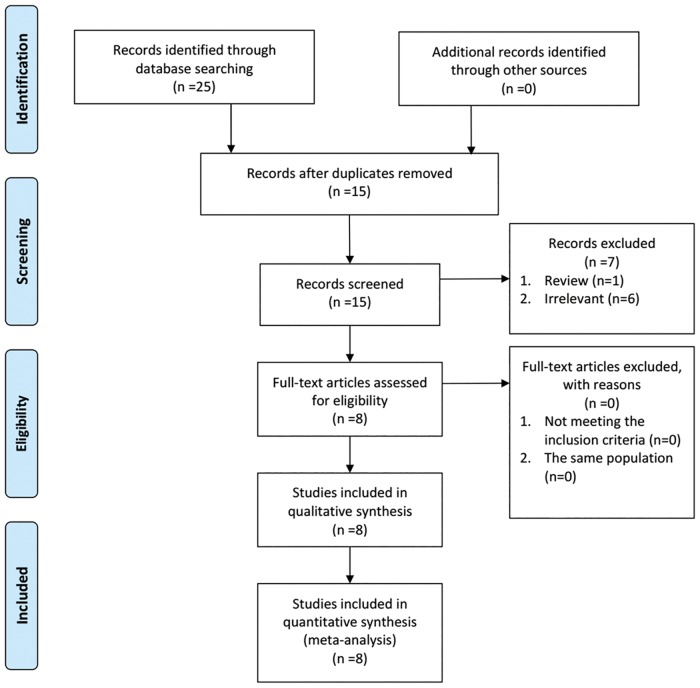
Flow diagram of study recruiting.

A flow diagram of the included and excluded studies was shown in Fig 1. According to the search strategy, 25 citations were identified from the four databases. After removing the duplicates (n = 10), two reviewers screened the titles and abstracts of potentially relevant studies (n = 15) independently. Finally, a total of 8 studies were included for meta-analysis [13–20].

### Characteristics and quality assessment of study

All included studies published from 2015 to 2016 were designed as case-control studies including 401 GDM patients and 421 healthy pregnant women. The characteristics of the studies included in the present meta-analysis were shown in Table 1. Of the 8 included studies, three were carried out in Turkey [13, 17, 20], two in China [18, 19], one in Austria [15], one in Poland [16] and one in German [14]. The sample size of these studies ranged from 21 to 97. Three of the included studies involved in the GDM women with BMI < 28 kg/m^2^ [14,20], and the rest five with BMI ≥ 28 kg/m^2^ [13, 15–18]. Blood samples for betatrophin measurement were collected in the second [13, 17, 19, 20] or third [14–16, 18] trimester of gestation. Except for the study conducted in German that used the American Diabetes Association criteria to carry out GDM diagnosis, all studies used the criteria suggested by International Association for Diabetes in Pregnancy Study Group to diagnose GDM.

**Table 1:** Characteristics of studies included in the meta-analysis.

Study	Country	Sample	Methods	Case group		Control group		Measurement trimester	Average age	Average BMI (kg/m^2^)
				Sample size	Betatrophin	Sample size	Betatrophin			
Erol, 2015	Turkey	Serum	ELISA	45	635.8 ± 258.60	45	320.1 ± 140.37	Second	29 ± 6.1	28.1 ± 5.7
Ebert, 2015	German	Serum	ELISA	74	1.79 ± 0.21	74	1.58 ± 0.18	Third	31 ± 7.5	24.5 ± 6.6
Yilmaz, 2015	Turkey	Plasma	ELISA	50	604.1 ± 122.60	60	420.1 ± 77.90	Second	31.84 ± 3.69	28.96 ± 3.51
Trebotic, 2015	Austria	Plasma	ELISA	21	29.24 ± 4.39	19	18.12 ± 8.65	Third	30.95 ± 5.15	29.57 ± 5.62
Natalia, 2015	Poland	Serum	ELISA	97	1.92 ± 0.2	93	1.63 ± 0.17	Third	31.5 ± 1.75	28.4 ± 1.85
Wang, 2016	China	Plasma	ELISA	30	765.35 ± 226.99	30	550.53±176.41	Third	29.03 ± 3.56	31.02 ± 4.66
Huang, 2016	China	Serum	ELISA	22	1024.3 ± 154.38	27	1513.6 ± 247.1	Second	30.8 ± 4.6	27.6 ± 3.5
Ersoy, 2016	Turkey	Serum	ELISA	62	1.8 ± 0.44	73	1.1 ± 0.35	Second	32.52 ± 3.92	26.2 ± 2.2

Note: ELISA: enzyme-linked immunosorbent assay; BMI: body mass index.

**Fig 2:**
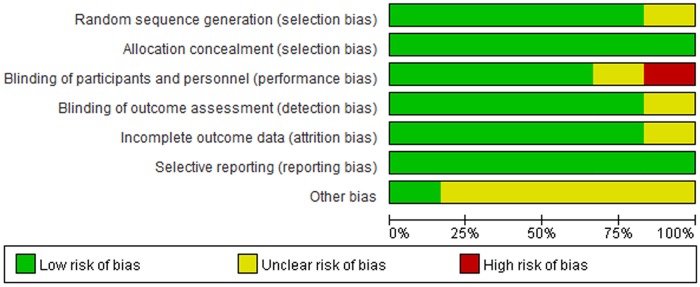
Risk of bias graph. The Cochrane collaboration’s tool was used to evaluate risk of bias.

The assessment on the quality of the included studies was shown in Fig 2.

### Overall meta-analysis

**Fig 3:**
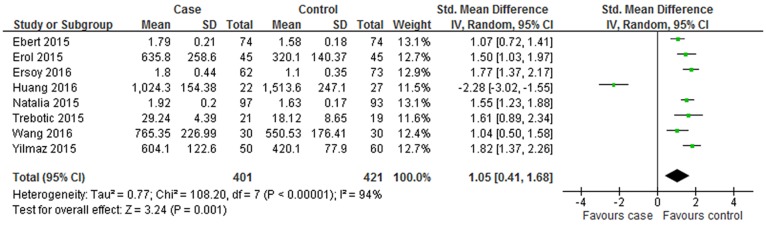
Forest plot of circulating betatrophin levels in GDM or healthy pregnant women. The random effect model (Inverse Variance method) was applied.

As indicated in Fig 3, the overall levels of circulating betatrophin in GDM patients were higher than that in the healthy controls with statistical significance (SMD = 1.05; 95% CI: 0.41–1.68, P = 0.001). The SMDs from the individual studies were analyzed using random-effects models, as the heterogeneity was considered significant (P < 0.001, I^2^ = 94%).

**Fig 4:**
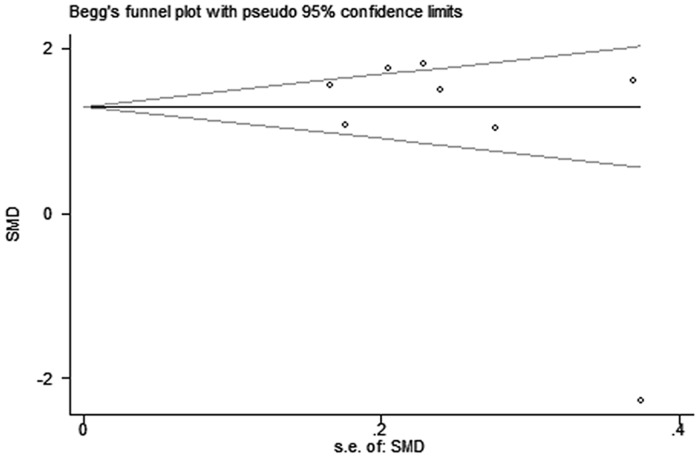
Begg’s funnel plot of included studies for potential publication bias.

**Fig 5:**
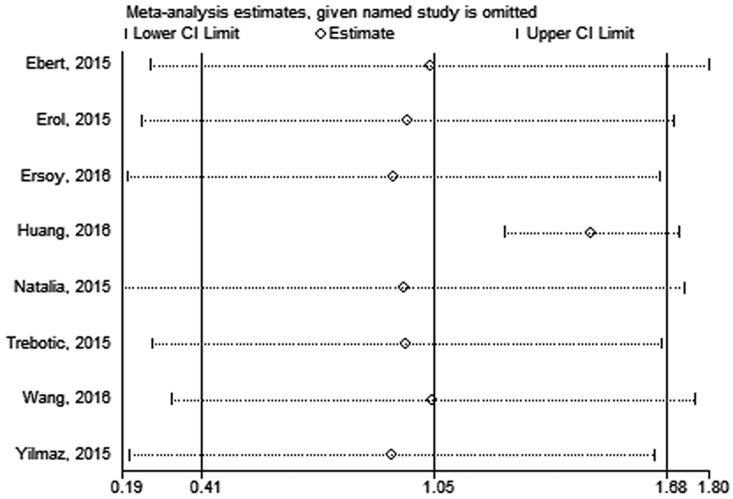
Sensitivity analysis of the circulating betatrophin levels in GDM or healthy pregnant women.

**Fig 6:**
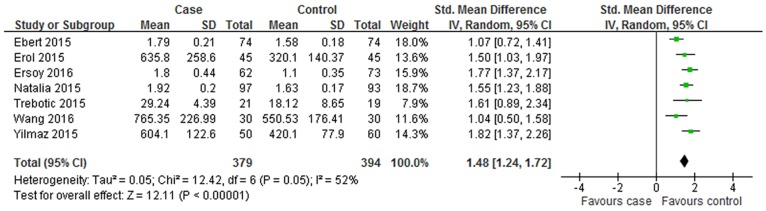
Forest plot of the circulating betatrophin levels in GDM or healthy pregnant women after the extraction of one study. The random effect model (Inverse Variance method) was applied.

No significant publication bias was found in our meta-analysis as indicated in Fig 4 (Begg’s test: P = 0.170; Egger’s test: P = 0.190). Sensitivity analysis was performed to explore potential sources of heterogeneity and assess relevant changes on the combined results. As suggested in Figs 5 and 6, the estimates effects indicated relevant changes on the combined results by study removals and the study carried out by Huang *et al* [19] may contribute to the methodological heterogeneity, ranging the heterogeneity from 94% (P < 0.001) for inclusion to 52% (P = 0.050) for exclusion. After strict screening again, we found that the study did meet the inclusion criteria.

### Subgroup analysis

**Fig 7:**
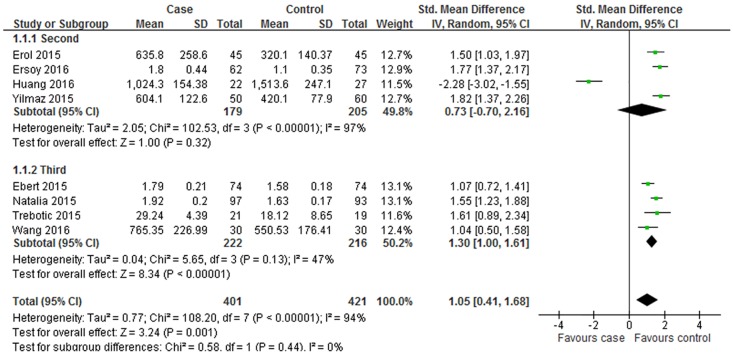
Subgroup analysis of circulating betatrophin levels in GDM or healthy pregnant women based on different trimester. The fixed effect model (Inverse Variance method) was applied.

**Fig 8:**
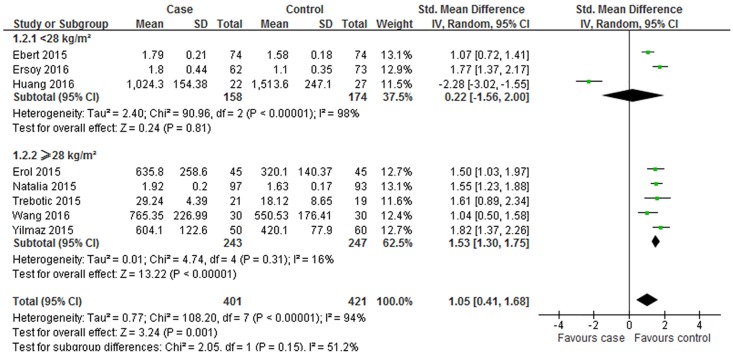
Subgroup analysis of circulating betatrophin levels in GDM or healthy pregnant women based on BMI. The fixed effect model (Inverse Variance method) was applied.

To investigate the possible sources of heterogeneity and obtain thorough information from this meta-analysis, subgroup analysis was further carried out. Subgroup analysis was conducted by geographic site, the trimester of betatrophin measurement, sample and mean of age and BMI in women with GDM. The comprehensive results were shown in Table 2 and Figs 7 and 8.

**Table 2:** Subgroup meta-analysis of the included studies.

Stratification	Study number	SMD	95% CI	*P* ^a^	Heterogeneity			
					*Tau*^*2*^	*Chi*^*2*^	*P* ^b^	*I*^*2*^
**Geographic site**								
Asian	2	-0.61	-3.87–2.65	0.710	5.43	51.07	<0.001	98%
European	6	1.53	1.29–1.78	<0.001	0.04	9.82	0.080	49%
**Measurement trimester**								
Second	4	0.73	-0.7–2.16	0.320	2.05	102.53	<0.001	97%
Third	4	1.3	1–1.61	<0.001	0.04	5.65	0.130	47%
**Average age**								
<30 years	2	1.29	0.84–1.74	<0.001	0.04	1.59	0.210	37%
≥30 years	6	0.96	0.12–1.8	0.020	1.02	106.61	<0.001	95%
**Average BMI**								
<28 kg/m^2^	3	0.22	-1.56–2	0.810	2.4	90.96	<0.001	98%
≥28 kg/m^2^	5	1.53	1.30–1.75	<0.001	0.01	4.74	0.310	16%
**Sample**								
Plasma	3	1.5	1–1.99	<0.001	0.11	4.71	0.090	58%
Serum	5	0.72	-0.18–1.63	0.120	1.01	95.6	<0.010	96%

P values in subgroups.

^b^P values for Cochran’s Q statistic test used to assess the heterogeneity.

BMI: body mass index; CI: confidential interval; SMD: standard mean difference.

When stratifying by the trimester of betatrophin measurement, these studies were classified as the second trimester and the third trimester. Four studies conducted in the second and the rest in third presented a conclusion of heterogeneity (second: P < 0.001, I^2^ = 97%; third: P = 0.130, I^2^ = 47%), and the random effect model was chosen to do the pooled analysis. The results in Fig 7 revealed that the circulating betatrophin levels measured in the third trimester were higher in women with GDM than that in controls (SMD = 1.3, 95% CI: 1–1.61, P < 0.001). However, the index of betatrophin levels in the second trimester demonstrated no statistical significance (SMD = 0.73; 95% CI: -0.7–2.16, P = 0.320).

In the subgroup analysis depending on BMI in Fig 8, the random effect model was chosen to do the pooled analysis because significant heterogeneity was observed (BMI < 28 kg/m^2^: P < 0.001, I^2^ = 98%; BMI ≥ 28 kg/m^2^: P = 0.310, I^2^ = 16%). For women with BMI ≥ 28 kg/m^2^, the circulating betatrophin levels increased in the GDM women (SMD = 1.53; 95% CI: 1.3–1.75, *P* < 0.001); however, for women with BMI < 28 kg/m^2^, the difference was not significant (SMD = 0.22; 95% CI: -1.56 to 2, P = 0.810).

When stratifying by geographic site, these studies were classified as the European group and the Asian group. For the European group, the betatrophin levels increased in the GDM patients (SMD = 1.53; 95% CI: 1.29–1.78, P < 0.010); however, for the Asian group, the difference was not significant (SMD = -0.61; 95% CI: -3.87–2.65, P = 0.710). The studies were classified to two subgroups according to the average age of the GDM cut-off of 30 years. The results indicated that both the two groups showed higher betatrophin levels in women with GDM (≥ 30 years: SMD = 0.96; 95% CI: 0.12–1.8, P = 0.020; < 30 years: SMD = 1.29; 95% CI: 0.84–1.74, P < 0.010). In the subgroup analysis of sample source of betatrophin, the difference of betatrophin levels between the GDM participants and controls was not statistically significant for the measurement of betatrophin from serum (SMD = 0.72; 95% CI: -0.18–1.63, P = 0.120); however, the difference was considered significant for the measurement of betatrophin from plasma (SMD = 1.5; 95% CI: 1–1.99, P < 0.010).

## Discussion

This systematic review and meta-analysis found that circulating betatrophin levels were significantly higher in women with GDM than healthy pregnant controls. The conclusion was also available in the subgroups of participants with BMI ≥ 28 kg/m^2^ and in the third trimester. The changes of betatrophin levels in GDM patients had been already suggested by other authors, however, to our knowledge no meta-analysis has been performed to date.

GDM is identified as any degree impairment of glucose tolerance with onset or first recognition during pregnancy. Pregnancy is typically accompanied by physiological insulin resistance that begins the second trimester and progresses through the third trimester, leading to an increase in maternal insulin secretion to maintain blood glucose levels as a consequence of adaptive pancreatic β-cell proliferation. Dysfunction in exacerbation of pancreatic β-cell or impairment of compensatory increases in insulin secretion from these cells or both leads to GDM [5]. The precise mechanisms of insulin resistance underlying GDM remain unknown. It is probably as a result of upregulation of insulin antagonist hormones.

In view of the prevalence of GDM, an increasing number of studies have involved in exploring the physiological and pathological mechanisms of GDM in animal models and human beings [23–28]. As a novel glucolipid metabolic regulation factor, betatrophin is getting more and more attention, which has been investigated in humans, particularly in the patients of DM and obesity. Previous studies focuses on the relationship between betatrophin and T2DM or obesity, while the focus shifts to GDM in recent two year. Several studies revealed that GDM women showed higher levels of betatrophin than control subjects [13–18, 20], indicating that augmented insulin resistance and enhanced insulin demand in GDM may contribute to the upregulation of betatrophin levels. In addition, Natalia *et al* and Wang *et al* suggested that not only maternal but also cord blood betatrophin levels were increased in the patients with GDM [16, 18, 29]. Furthermore, the three studies mentioned above found an interesting phenomenon that betatrophin concentration in cord blood was higher than that in maternal serum, which might suggest its role in promoting β-cell proliferation during intrauterine life. The alteration of betatrophin has been reported to be influenced by multiple factors, such as age, sex, duration of diabetes and BMI, as well as environmental and genetic factors [11, 12, 18]. Fu *et al* indicated that betatrophin levels were elevated in obesity and were positively correlated with BMI [6]. However, Fenzl *et al* revealed that circulating betatrophin did not correlate with BMI [30, 31]. While in GDM patients, Yilmaz *et al* demonstrated that betatrophin levels increased with age and BMI, demonstrating that obesity and old age may be contributing factors for increased betatrophin levels in GDM [17]. Taken together, current population-based studies indicated that circulating betatrophin levels could be a biomarker candidate of GDM. Conversely, Huang *et al* suggested that betatrophin levels were much lower in women with GDM than that in the corresponding controls, although the difference did not reach statistical significance [19]. However, the potential mechenisms are not clear because of the insufficient studies.

The current meta-analysis showed that the pooled value of mean [95% CI] was of statistical significance, revealing increased circulating levels of betatrophin in GDM. However, according to the over all forest plot in Fig 3, substantial heterogeneity (I^2^ = 94%) was observed among the studies. To find the sources of heterogeneity, subgroup analysis and sensitivity analysis was performed. Subgroup analysis was depended on geographic site, sample source, the trimester of betatrophin measurement and group mean of age and BMI in women with GDM. In the subgroup of Europe participants, the betatrophin levels of GDM patients were relatively higher than that in healthy pregnant women, while the trend was not available in the subgroup analysis of Asia patients. The dietary habits in different locations may explain the results. Western eating habits are more likely to cause obesity and insulin resistance. Previous research indicated that the circulating betatrophin levels were associated with the BMI and age [6, 12]. In our meta-analysis, subgroup analysis indicated that betatrophin levels were elevated in GDM patients with BMI ≥ 28 kg/m^2^ and during the third trimester, while not in women with BMI < 28 kg/m^2^ or during the second trimester. And the betatrophin levels in GDM participants were higher than women with normal glucose tolerance regardless of age. The higher tendency of insulin resistance and increasing level of lipid profiles in the third trimester and in obesity patients may contribute to the above results. When the subgroup analysis of different countries, BMI, age and measurement time was carried out, we found that the heterogeneity was decreased. The results indicated that the source of heterogeneity may be partly from clinical heterogeneity. Additionally, sensitivity analysis was performed by excluding articles one by one before reanalyzing statistically. The results showed that the final meta-analysis results were stable with remove of any study, which provided more credibility to our interpretation of results. More important, the results of sensitivity analysis indicated that the study performed by Huang *et al* [19] probably contributed to the methodological heterogeneity.

Giving the acute adverse outcomes and long-term effects on pregnant women and offspring, GDM is drawing increasing attention in recent years. The topic is therefore of relevance from the public health perspective and the present meta-analysis can contribute to clarify some of pathophysiological mechanisms of GDM by providing statistical assessment. However, there are some limitations to this meta-analysis. First, the publication bias cannot be avoided absolutely, as only published studies in English and Chinese in the selected databases were included. Second, the absolute value of betatrophin was widely different among included studies. Third, only fasting circulating betatrophin was detected. As a food intake-induced hormone, postprandial betatrophin after standard diet could be more meaningful in further investigation. Fourth, we have no access to get the original data of the included literature, so we cannot guarantee the accuracy of the data. Therefore, the results should be interpreted with caution.

In conclusion, the current meta-analysis revealed increased circulating levels of betatrophin in patients with GDM. Women with GDM generally have few obvious related symptoms, then betatrophin might be a potential predictor for assessing GDM. This result could help clinical staff to instruct women with GDM to prevent the progression of GDM. However, the regulation and metabolism of betatrophin remained unclear in human. For further studies, well-designed epidemiological studies with large sample sizes and strict stratification of potential confounding factors should be performed. It will be meaningful and interesting to explore the potential role of betatrophin in GDM prediction and therapeutics.

## Supporting Information

S1 Table PRISMA 2009 checklist. (DOC)
