# Correction: Circulating Betatrophin Levels and Gestational Diabetes Mellitus: A Systematic Review and Meta-Analysis

**DOI:** 10.1371/journal.pone.0172449

**Published:** 2017-02-14

**Authors:** The PLOS ONE Staff

The fifth sentence of the first paragraph under the subheading “Characteristics and quality assessment of study” in the Results section should have cited reference 19 in addition to references 14 and 20. The publisher apologizes for the error.

The correct sentence should read: “Three of the included studies involved in the GDM women with BMI < 28 kg/m^2^ [14, 19, 20], and the rest five with BMI ≥ 28 kg/m^2^ [13, 15–18].”

Reference 19 is: Huang Y, Fang C, Ma Z, Guo H, Wang R, Hu J. Betatrophin Levels were Increased in Pregnant Women with or without Gestational Diabetes Mellitus and Associated with Beta Cell Function. Rev Bras Ginecol Obstet. 2016;38(6):287–92. doi: 10.1055/s-0036-1584566. pmid:27399923
